# L-Endoglin Overexpression Increases Renal Fibrosis after Unilateral Ureteral Obstruction

**DOI:** 10.1371/journal.pone.0110365

**Published:** 2014-10-14

**Authors:** Bárbara Oujo, José M. Muñoz-Félix, Miguel Arévalo, Elena Núñez-Gómez, Lucía Pérez-Roque, Miguel Pericacho, María González-Núñez, Carmen Langa, Carlos Martínez-Salgado, Fernando Perez-Barriocanal, Carmelo Bernabeu, José M. Lopez-Novoa

**Affiliations:** 1 Renal and Cardiovascular Research Unit, Department of Physiology and Pharmacology, University of Salamanca, Salamanca, Spain; 2 Biomedical Research Institute of Salamanca (IBSAL), Salamanca, Spain; 3 Institute Queen Sophie for Renal Research, Salamanca, Spain; 4 Department of Human Anatomy and Histology, University of Salamanca, Salamanca, Spain; 5 Centro de Investigaciones Biológicas, Consejo Superior de Investigaciones Científicas, CSIC, and Centro de Investigación Biomédica en Red de Enfermedades Raras (CIBERER), Madrid, Spain; 6 Health Sciences Studies Institute of Castilla y León (IESCYL), Salamanca, Spain; University of Southern California, United States of America

## Abstract

Transforming growth factor-β (TGF-β) plays a pivotal role in renal fibrosis. Endoglin, a 180 KDa membrane glycoprotein, is a TGF-β co-receptor overexpressed in several models of chronic kidney disease, but its function in renal fibrosis remains uncertain. Two membrane isoforms generated by alternative splicing have been described, L-Endoglin (long) and S-Endoglin (short) that differ from each other in their cytoplasmic tails, being L-Endoglin the most abundant isoform. The aim of this study was to assess the effect of L-Endoglin overexpression in renal tubulo-interstitial fibrosis. For this purpose, a transgenic mouse which ubiquitously overexpresses human L-Endoglin (*L-ENG^+^)* was generated and unilateral ureteral obstruction (UUO) was performed in *L-ENG^+^* mice and their wild type (WT) littermates. Obstructed kidneys from *L-ENG^+^* mice showed higher amounts of type I collagen and fibronectin but similar levels of α-smooth muscle actin (α-SMA) than obstructed kidneys from WT mice. Smad1 and Smad3 phosphorylation were significantly higher in obstructed kidneys from *L-ENG^+^* than in WT mice. Our results suggest that the higher increase of renal fibrosis observed in *L-ENG^+^* mice is not due to a major abundance of myofibroblasts, as similar levels of α-SMA were observed in both *L-ENG^+^* and *WT* mice, but to the higher collagen and fibronectin synthesis by these fibroblasts. Furthermore, *in vivo* L-Endoglin overexpression potentiates Smad1 and Smad3 pathways and this effect is associated with higher renal fibrosis development.

## Introduction

Chronic kidney disease (CKD) is a pathology that affects nearly 10% of the population. This illness is characterized by a progressive decrease in glomerular filtration rate that eventually leads to renal failure. Tubulointerstitial fibrosis, a major determinant of CKD, occurs by myofibroblast activation and proliferation in the renal interstitium with accumulation of extracellular matrix (ECM) [1].

One of the most important molecules related with renal fibrosis is transforming growth factor β (TGF-β) being TGF-β1 the most studied isoform [2], [3]. Two receptors, type I (TβRI) and type II (TβRII) are necessary for TGF-β signaling. Whereas there is only one type II receptor for TGF-β1, two type I receptors have been described, Activin Like Kinase Receptor 1 and 5 (ALK1 and ALK5, respectively), which phosphorylate the specific Smads. Thus, TGF-β is able to signal through ALK1-Smad1/5 or ALK5-Smad2/3 pathways, resulting in different cell responses [4]. It is traditionally accepted that TGF-β1 exerts its profibrotic effects through the ALK5 pathway [5]. However, Smad1 pathway activation has been recently associated with renal fibrosis in diabetic and obstructive nephropathy [6], [7]. Endoglin (Eng), a 180 KDa-homodimeric transmembrane glycoprotein, acts as co-receptor of some members of the TGF-β superfamily [8]. *In vitro* experiments have shown that endoglin antagonizes the profibrotic effect of TGF-β [9], [10]. It has been also observed that endoglin is overexpressed in several experimental models of renal fibrosis such as renal mass reduction [11], unilateral ureteral obstruction (UUO) [12] and in radiation-induced fibrosis [13]. Moreover, endoglin involvement has been studied in other fibrotic pathologies such as scleroderma [14], hepatic fibrosis [15] or cardiac fibrosis [16], [17].

Endoglin presents two membrane isoforms called L-Endoglin (long) and S-Endoglin (short). These isoforms, described in human and mice, are produced by alternative splicing and differ from each other in the length of their cytosolic tails (shorter in S-Endoglin) [18], [19]. As L-Endoglin is the most abundant isoform [8], we have focused our study on the effect of the overexpression of this isoform in renal fibrosis.

The unilateral ureteral obstruction (UUO) model of obstructive nephropathy reproduces the most representative features of tubulointerstitial fibrosis: extracellular matrix (ECM) accumulation, tubular apoptosis, myofibroblast activation and proliferation, tubular deletion and inflammatory cell infiltration, and it has been used in multiple studies to assess the mechanisms involved in these processes [20]. The aim of the present study was to elucidate the effect of L-Endoglin overexpression in renal fibrosis induced by UUO.

## Materials and Methods

### Generation and characterization of *L-ENG* ^+^ transgenic mice

**Figure 1:**
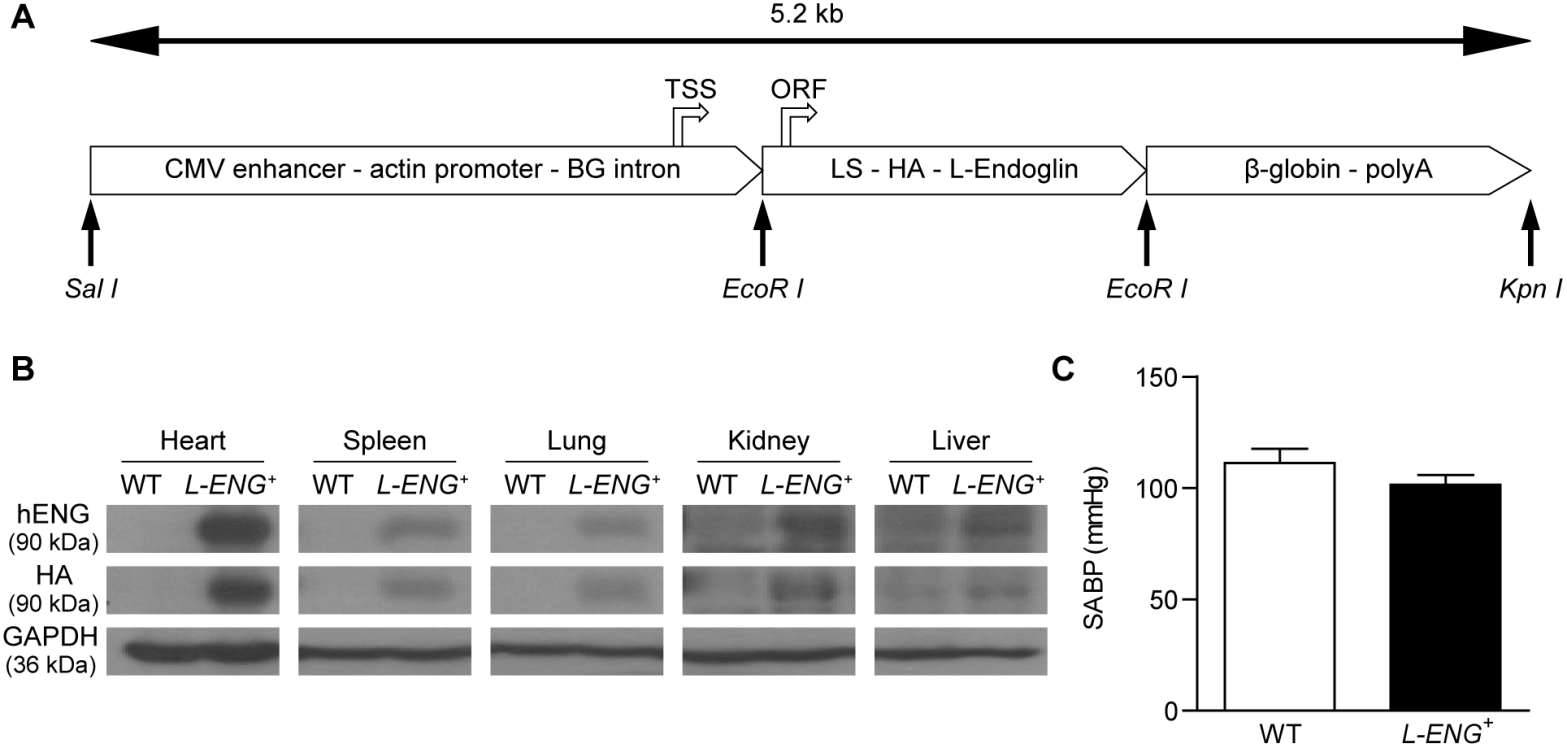
Transgenic mice expressing human L-endoglin. (a) Schematic representation of the DNA construct used to generate transgenic mice expressing human L-endoglin. A 5.2-kb *SalI/KpnI* fragment, containing a CMV enhancer, an actin enhancer/promoter, a β-globin (BG) intron (fragment *SalI/EcoRI*), the endoglin leader sequence-LS, the hemagglutinin epitope-HA, the human L-endoglin cDNA (fragment *EcoRI*/*EcoRI*) and a β-globin polyadenylation site-polyA (fragment *EcoRI*/*KpnI*), was microinjected in the pronuclei of fertilized oocytes to generate *L-ENG* ^+^ mice. The transcription start site (TSS) and the translation initiation of the open reading frame (ORF) are indicated. (b) Expression of human L-endoglin protein in different tissues from *L-ENG* ^+^ mice. Protein extracts were analyzed by western blot using anti-endoglin and anti-HA antibodies. (c) Effects of human L-endoglin overexpression in systolic arterial blood pressure (SABP). N = 12 mice per group.

To generate a transgenic mice expressing human L-endoglin (*L-ENG* ^+^), HA-tagged L-endoglin cDNA in pDisplay [21] and L-endoglin cDNA in pCEXV [18] were used to derive by PCR a construct encoding the endoglin leader sequence (amino acids 1–25) followed by the influenza hemagglutinin epitope (HA; Tyr-Pro-Tyr-Asp-Val-Pro-Asp-Tyr-Ala) and the mature L-endoglin (variant 1, amino acids 26–658). The resulting 2.3-kb cDNA fragment was cloned into the *EcoRI* site of pcEXV vector [18]. The L-endoglin-pcEXV vector was digested with *EcoRI*, and the endoglin fragment was inserted into the *EcoRI* site of the pCAGGS plasmid [22] under the control of a ubiquitous actin promoter. The pCAGGS plasmid contains, from 5′ to 3′, a CMV enhancer, a chicken enhancer and promoter, a β-globin intron, an *EcoRI* site, and a β-globin polyadenylation motif [23]. To obtain linear DNA fragments for microinjection, the expression vector L-endoglin-pCAGGS was digested with *SalI*/*KpnI* and the endoglin containing 5.2-kb fragment was separated by agarose gel electrophoresis. The purified fragment (Figure 1a) was microinjected into CBAxC57BL/6J fertilized eggs at the University of Salamanca Transgenic Facility, using standard protocols. Progeny was screened for endoglin transgene by PCR of tail DNA using forward (5′-AGAGCATCCTCCTCCGACTGG-3′) and reverse (5′-TGAAGCCACGAATGTTTTTCT-3′) primers. *L-ENG* ^+^ transgenic founders were crossed with C57BL/6J mice to perpetuate the transgenic lines. Three independent transgenic founders were generated and similar phenotypic features were observed in all the derived transgenic lines. Mice were kept in a pathogen-free facility for genetically modified animals (Servicio de Experimentación Animal, SEA; University of Salamanca).

### Mice and animal disease

A breeding colony of adult *L-ENG* ^+^ animals has been maintained in our facilities for more than 2 years. Studies were performed in the 6^th^–7^th^ generation of backcrossing. All studies were performed in parallel in *L-ENG* ^+^ and WT male mice aged 8 weeks. The UUO procedure was performed as previously described [12], [24]. In brief, mice were anesthetized with isofluorane (Schering-Plough, Madrid, Spain), the abdomen was opened and the left ureter was located and occluded in two places with non-absorbable 5-0 silk. The abdomen was closed with running suture and the skin was closed with interrupted sutures. After surgery, animals were kept warm during 3 hours and a single dose of analgesic (buprenorphine, Schering-Ploug, Madrid, Spain) was injected subcutaneously to enhance the post-operation phase. Sham-operated (SO) animals were included as controls. Subsequently, mice were housed in a temperature-controlled room with a 12 h light/dark cycle and fed on standard chow (Panlab, Barcelona, Spain) and water *ad libitum*. UUO was maintained during 15 days.

All the studies were approved by the Animal Care and Use Committees of the University of Salamanca and mice were cared in compliance with the rules of European Union and with the US Department of Health and Human Services Guide for the Care and Use of Laboratory Animals.

### Blood pressure measurement

Before performing UUO, blood pressure was recorded in conscious *L-ENG^+^* and WT mice (n = 12 in each group) with an automated multichannel system by using the tail-cuff method and a photoelectric sensor (Niprem 546; Cibertec, Madrid, Spain). Animals were previously accustomed for several days and measures were collected at the same hour during at least 3 days.

### Kidney sampling

Euthanasia was performed with i.p. pentobarbital overdose. Animals were perfused from the heart with heparinized saline solution. For histological studies, obstructed (O) and non obstructed (NO) kidneys were removed [WT: SO (n = 3); NO (n = 4); O (n = 4) and *L-ENG* ^+^: SO (n = 3); NO (n = 4); O (n = 4)], halved, and fixed in 4% buffered formalin for 24 h. Subsequently, kidneys were dehydrated in ascending series of ethanol, cleared in xylene and embedded in paraffin. Kidney sections (3 µm) were stained with either hematoxylin-eosin or Masson’s trichrome for light microscopy analysis, as previously described [12], [24]. The other half was immediately frozen used for protein and mRNA obtention.

### Morphometric studies

For image analysis of fibrosis, additional 5-µm thick renal sections were cut, mounted on glass slides and stained with Sirius red (Direct Red 80, Sigma-Aldrich Quimica, Madrid, Spain) to evaluate the area occupied by collagen fibers. From each kidney, a total of 15 interstitial images of each slide were captured and processed with a high-resolution video camera (Sony, DF-W-X710) connected to a light microscope (Nikon Eclipse 50i) using the 20x objective and a green optical filter (IF 550). The area occupied by collagen was measured using a computerized image analysis system (Fibrosis HRR, Master Diagnostic), as described [25]. The values obtained were expressed in square micrometers. Immunohistochemistry studies were quantified by using ImageJ software (Rasband, W.S., ImageJ, National Institutes of Health, Bethesda, Maryland, USA). Ten images per slide were digitalized and processed with the same video camera and microscope described for the analysis of fibrosis.

### Immunohistochemical studies

Three µm sections were processed for immunohistochemistry as follows. Endogenous peroxidase was blocked with 3% hydrogen peroxide and immunohistochemical staining for fibronectin (F3648, 1∶100 dilution; Sigma-Aldrich, St Louis, MO, USA), collagen I (Ab21286, 1∶800; Abcam, MA, USA), α-SMA (A2547, 1∶1,000; Sigma-Aldrich, St Louis, MO, USA and NCL-MSA 1∶200; Leica Microsistemas, Barcelona, Spain), phospho-Smad2/3 (sc-11769, 1∶50 dilution; Santa Cruz Biotechnology Inc, CA, USA) and phospho-Smad1 (Ab63439, 1∶50 dilution; Abcam, Cambridge, UK) were performed. Then, sections were washed three times in PBS and incubated with the Novolink Polymer Detection System (Novocastra, MA, USA), followed by reaction with 3,3′diaminobenzidine as chromogen. Negative WT slides were prepared without primary antibody.

### Western blot and PCR analysis

For western blot, after euthanasia with i.p. pentobarbital overdose, SO, NO and O kidneys were removed from wild type and *L-ENG* ^+^ mice [WT: SO (n = 5); NO (n = 7); O (n = 7) and *L-ENG^+^*: SO (n = 5); NO (n = 7); O (n = 7)] and immediately stored at −80°C. Tissue protein extracts were prepared as previously described [12]. Total kidney protein lysates were fractioned by SDS-PAGE, transferred onto PVDF membrane (Millipore Billerica, MA, USA) and incubated overnight at 4°C with the following antibodies: anti-fibronectin (AB2033, 1∶1,000 dilution; Chemicon International, Temecula, CA, USA), anti-collagen type I (Ab765P, 1∶1,000 dilution; Chemicon International, Temecula, CA, USA), anti-α-SMA (A2547, 1∶1,000; Sigma-Aldrich, St Louis, MO, USA), anti-Smad1 (sc-7965, 1∶1,000 dilution; Santa Cruz Biotechnology, CA, USA), Smad2/3 (sc-6032, 1∶1,000 dilution; Signaling Technology, MA, USA), anti-phospho-Smad1 (06-702, 1,1000, 1∶1,000 dilution; Abcam, Cambridge, UK), anti-phospho-Smad2 (566415, 1∶1,000 dilution; Calbiochem, La Jolla, CA, USA), anti-phospho-Smad3 (9520, 1∶1,000 dilution; Cell Signaling Technology Inc., Danvers, MA, USA), anti-GAPDH (AM4300, 1∶40,000 dilution; Invitrogen, CA, USA), and anti-endoglin (human) (sc-20632, 1∶1,000 dilution; Santa Cruz Biotechnology, CA, USA). Membranes were washed and incubated with the corresponding secondary antibodies (1∶10,000 dilution; Bio-Rad, St Louis, MO, USA) for 45 minutes. Development was performed using a chemiluminescent reagent (ECL detection reagent, Amersham Biosciences) and signals were recorded on X-ray film (Fujifilm), followed by densitometric analysis using ImageJ software (Rasband, W.S., ImageJ, National Institutes of Health, Bethesda, Maryland, USA).

Total protein normalization to GAPDH was not performed since this loading control varies after UUO, as it has been described by other authors [26], [27], [28]. However, we have included the GAPDH blot in order to demonstrate that the increases in protein expression observed after UUO and the higher increased expression observed in *L-ENG* ^+^ animals is not due to an increase of housekeeping proteins.

For RT-PCR analysis, total RNA was isolated using Nucleospin RNAII (Macherey-Nagel), according to the manufacturer’s instructions. Single-strand cDNA was generated from 2 µg of total RNA using poly-dT as primer with M-MLV reverse transcriptase (Promega). Quantitative RT-PCR was performed in triplicate. Each 20 µl reaction contained 1 µl of cDNA, 400 nM of each primer, and 1x iQ SybrGreen Supermix (Bio-Rad). Standard curves were run for each transcript to ensure exponential amplification and to rule out non-specific amplification. Primers used were: For mouse Collagen Iα: forward 5′-GGAGAGAGCATGACCGATGGA-3′ and reverse 5′-GGTGGACATTAGGCGAGGAA-3. For mouse Fibronectin: forward 5′-TGACAGTTGGTCACCCTGTT-3′ and reverse 5′-GGTGTCTGGGTGACTTTCCT -3′. For mouse PRS13 (120 bp): forward 5′-GATGCTAAATTCCGCCTGAT-3′ and reverse 5′-TAGAGCAGAGGCTGTGGATG-3′. Cycling conditions for Fibronectin and PRS13: 95°C, 5 min, 35 cycles of 1 min 95°C, 1 min 59°C and 1 min 72°C, and an elongation cycle of 5 min 72°C. Cycling conditions for Collagen Iα: 95°C, 5 min, 35 cycles of 1 min 95°C, 1 min 56°C and 1 min 72°C, and an elongation cycle of 5 min 72°C. Gene expression was normalized to RPS13 expression. The reactions were run on an iQ5 Real-time PCR detection system (Bio-Rad).

### Renal fibroblast culture

Renal fibroblasts were obtained from a pool of animals (3 per group) in which UUO was maintained during 3 days, as previously described [7]. In brief, kidneys were removed and washed with saline solution (NaCl 0.9%) and Dulbecco solution (2.6 mM KCl, 1.5 mM KH_2_PO_4_, 137 mM NaCl, 8 mM Na_2_HPO_4_, 5.6 mM glucose) supplemented with 500 U/mL penicillin/streptomycin. Kidneys were cut in thin slices (∼0.2 mm) and embedded in a 0.45 mg/L collagenase type IA solution (Sigma-Aldrich, St Louis, MO, USA) during 40 minutes at 37°C. Tissue culture supernatants containing fibroblasts were placed in Dulbecco’s modified Eagle’s medium (Gibco, Life Technologies, Carlsbad) containing 10% fetal calf serum and 100 U/mL penicillin/streptomycin at 37°C in the presence of 5% CO_2_. In the third passage, when cultures achieved 80–90% confluence, cells were serum starved for 24 h before performing the experiments. Cells were treated with human TGF-β1 1 ng/mL (R&D Systems, Minneapolis, MN, USA) for 30 minutes in order to analyze Smad signaling as it has been described [29] or during 24 hours to analyze protein expression, as previously described [30], [31].

### Cell immunofluorescence

Fibroblasts seeded in cover slips were fixed with 4% formalin washed with PBS, permeabilized with 0.1% Triton X-100, blocked for 30 min with 2% BSA in PBS, treated with PBS-0.05% Tween 20 for 10 min, and incubated during 2 hours with anti α-SMA antibody or Oregon Green (Invitrogen) for total actin detection. Then, cells were incubated for 30 min with goat anti-mouse Cy3 (1∶1,000 dilution; Jackson Immuno-Research, West Grove PA, USA) in PBS in a dark chamber. For nuclei staining, incubation with 2 µM Hoechst 33258 (Molecular Probes, Barcelona, Spain) for 5 min in a dark chamber was performed. Cover slips were mounted on slides using Prolong gold antifade (Molecular Probes). Confocal images were obtained as previously described [24].

### Statistical analysis

Data are expressed as mean ± standard error of the mean (SEM). Comparison of means was performed by one way analysis of variance (ANOVA). Data was analyzed using the GraphPad Prism 5. A p-value lower than 0.05 was considered statistically significant.

## Results

### Characterization of *L-ENG* ^+^ transgenic mice

A transgenic mice expressing human L-endoglin (*L-ENG^+^*) under the control of a ubiquitous actin promoter was generated (Figure 1a). Protein levels of human L-Endoglin were evaluated in several tissues as lung, liver, spleen and heart by Western blot analysis (Figure 1b). Our results show that human L-Endoglin is expressed in all organs studied being this expression higher in heart and spleen. Human L-Endoglin band appears also in human tissues, as expected (Figure S1). Mice of three independent founder lines appeared healthy and were of the same size and weight as their non-transgenic littermates. They were fertile, showing a normal ratio of male to female littermates, had a normal lifespan, and they did not show notable changes in microscopic structures of the major organs (data not shown). Haemodynamic studies revealed that WT and *L-ENG* ^+^ mice have a similar systolic arterial blood pressure (Figure 1c), thus discarding hypertension as a possible risk factor to develop renal damage in these animals.

### Effects of L-ENG overexpression on renal collagen and fibronectin after UUO

**Figure 2:**
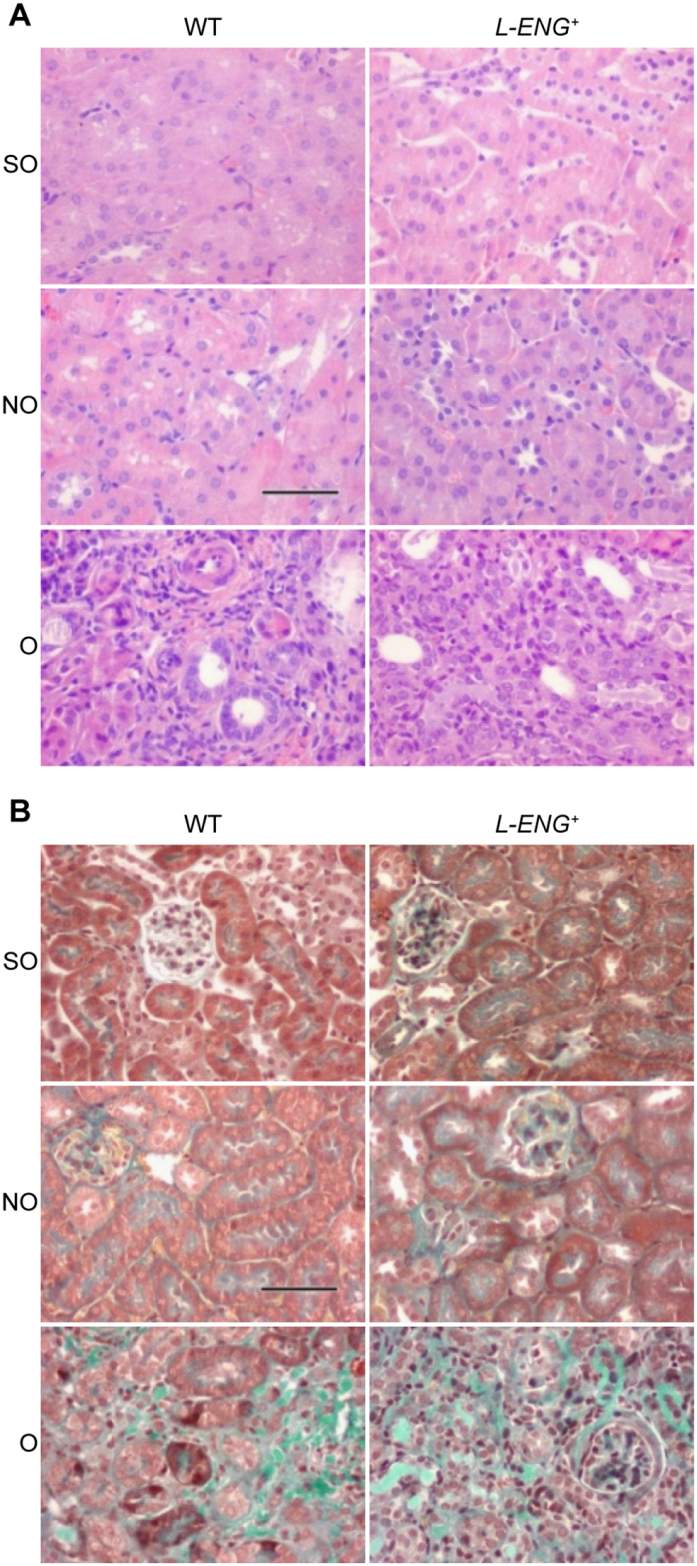
Effect of L-Endoglin overexpression on interstitial fibrosis after unilateral ureteral obstruction (I). Representative images of haematoxylin-eosin (a) and Masson’s trichrome (b) staining in sham operated (SO), non-obstructed (NO) and obstructed (O) kidneys from WT and *L-ENG* ^+^ mice. In O kidneys tubular dilatation, inflammatory cell infiltration and interstitial fibrosis can be observed. Bar = 100 µm.

**Figure 3:**
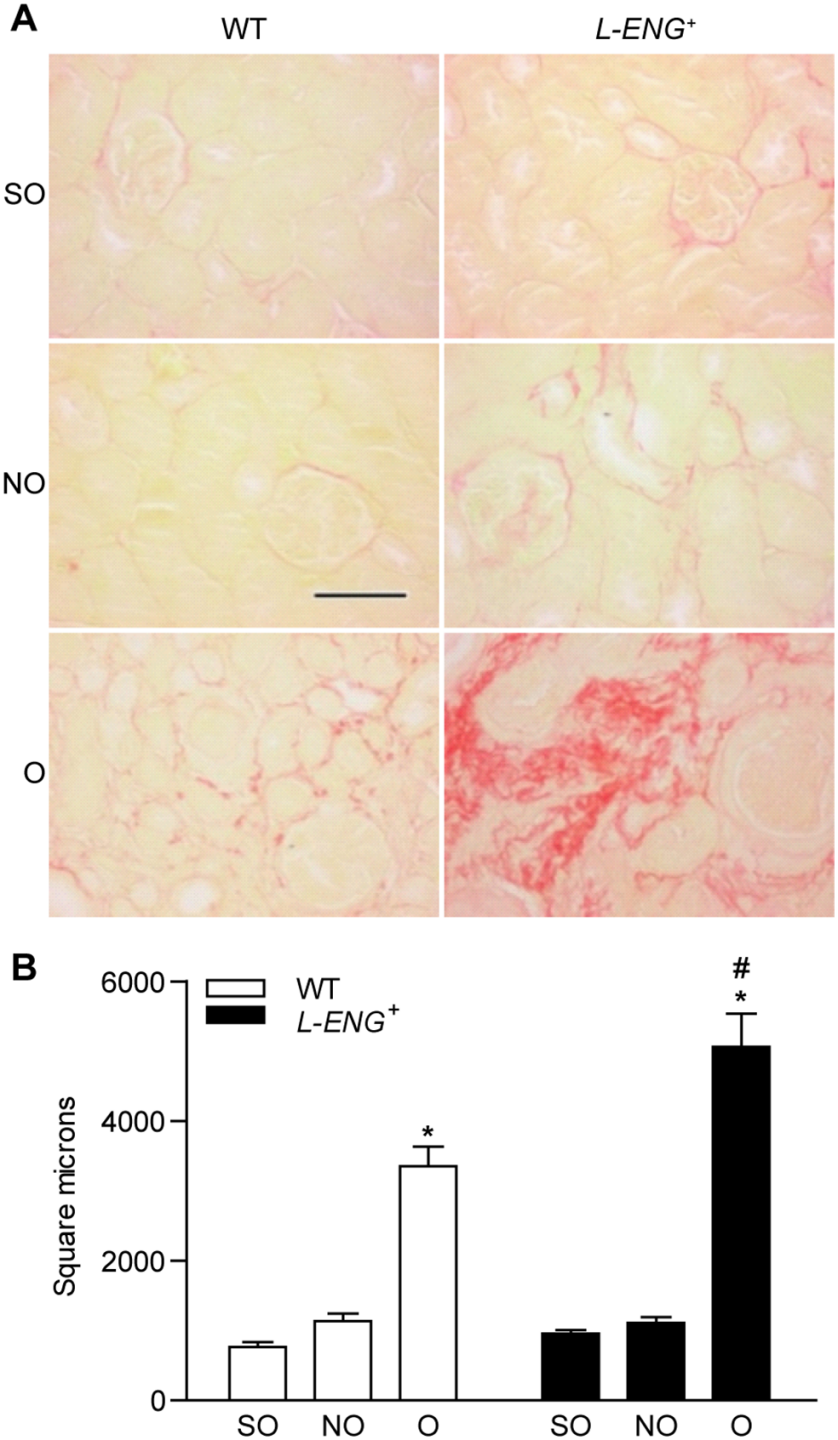
Effect of L-Endoglin overexpression on interstitial fibrosis after unilateral ureteral obstruction (II). (a) Representative images of Sirius Red staining in sham operated (SO), non-obstructed (NO) and obstructed (O) kidneys from WT and *L-ENG* ^+^ mice. Bar = 100 µm. (b) Morphometric quantification (mean ± SEM) of area stained by Sirius Red; SO (n = 3); NO (n = 4); O (n = 4) in each group of mice. *P<0.05 vs. their respective SO kidneys. ^#^P<0.05 vs. O kidneys from WT mice.

After 15 days of UUO, obstructed kidneys in both, wild type and *L-ENG* ^+^ mice, exhibited a severe hydronephrosis and the typical features of obstructive nephropathy. Thus, both hematoxylin-eosin and Masson’s trichrome staining revealed that the total thickness of the cortex and medulla was diminished and medullar compression was evidenced (Figure S2). In the cortex, tubules showed changes ranging from a dilated lumen with a flattened epithelium to necrosis of varying degrees. In most cases, an interstitial inflammatory infiltrate was observed, mainly in perivascular areas (Figure 2a). Fibrosis, detected with Masson’s trichrome staining, was irregularly distributed and more pronounced around blood vessels. Little or no tissue fibrosis was observed in non obstructed (NO) kidneys as compared with obstructed (O) kidneys. Obstructed kidneys from *L-ENG* ^+^ mice had a high degree of fibrosis, according to Masson’s staining, than WT mice. Sham-operated animals had normal kidney histology (Figure 2b). Sirius red staining showed that O kidneys from *L-ENG* ^+^ mice had a high degree of fibrosis compared to O kidneys from WT mice (Figure 3a, b). Morphometric studies performed in Sirius red-stained slides demonstrated that O kidneys from *L-ENG* ^+^ mice had more renal area occupied by fibrosis than O kidneys from WT mice (Figure 3b).

**Figure 4:**
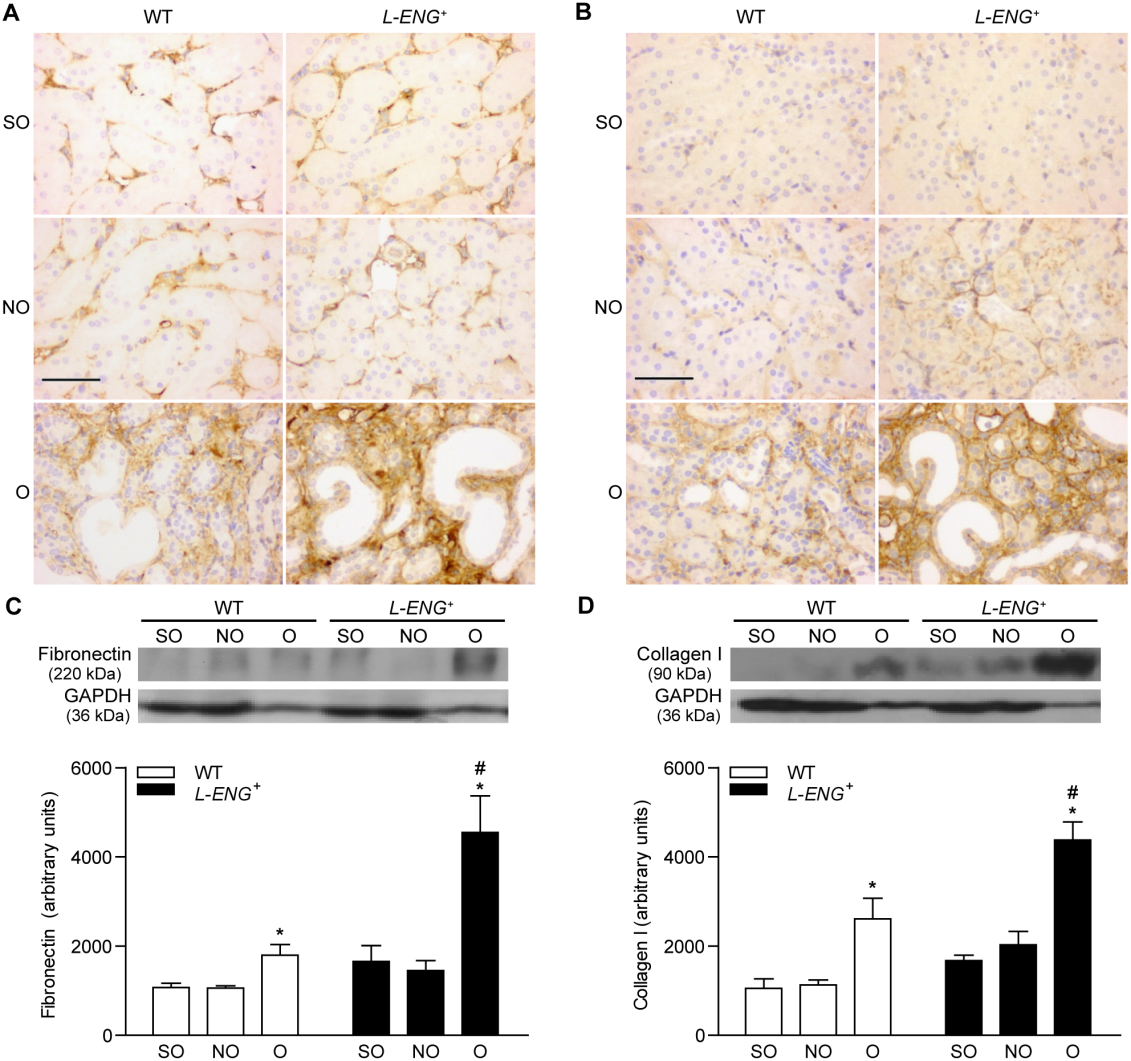
Effect of L-Endoglin overexpression on fibronectin and collagen I expression following ureteral obstruction. Representative immunohistochemistry images for fibronectin (a) and collagen I (b) in sham operated (SO), non-obstructed (NO) and obstructed (O) kidneys from WT and *L-ENG* ^+^ mice. Bar = 100 µm. Western blot analysis of fibronectin (c) and collagen I (d) protein amount in SO, NO and O kidneys from WT and *L-ENG^+^* mice. A representative western blot among 5–7 performed in each group is shown on top. Densitometry analysis is represented as the mean ± SEM of the 5–7 western performed per group. *P<0.01 vs. SO kidneys. ^#^P<0.05 vs. O kidneys from WT mice.

Both collagen I and fibronectin abundance, assessed by immunohistochemistry and western blot, showed an increased amount of these proteins in O respect to those of NO kidneys (Figure 4). Immunohistochemistry studies revealed that kidneys from sham-operated animals and NO kidneys from WT mice showed a weak expression of fibronectin relegated to the basement membranes. Collagen I was practically absent in kidneys from sham animals and in NO kidneys from WT, but was slightly present in NO kidneys from *L-ENG* ^+^ mice (Figure 4a). Staining for collagen I and fibronectin proteins in the interstitial space of O kidneys from *L-ENG* ^+^ mice was higher than in O kidneys from WT animals (Figure 4a, b). Similar results were obtained after western blot analysis. Thus, the amount of collagen-I and fibronectin in O kidneys from *L-ENG* ^+^ mice was significantly higher than in O kidneys from WT animals (Figure 4c, d).

Moreover, qPCR analysis showed an increase in both collagen I and fibronectin mRNA expression after UUO. This increase was slightly higher in *L-ENG^+^* than in WT mice but the difference was not statistically significant (Figure S3).

### Effects of L-ENG overexpression on myofibroblast abundance after UUO

**Figure 5:**
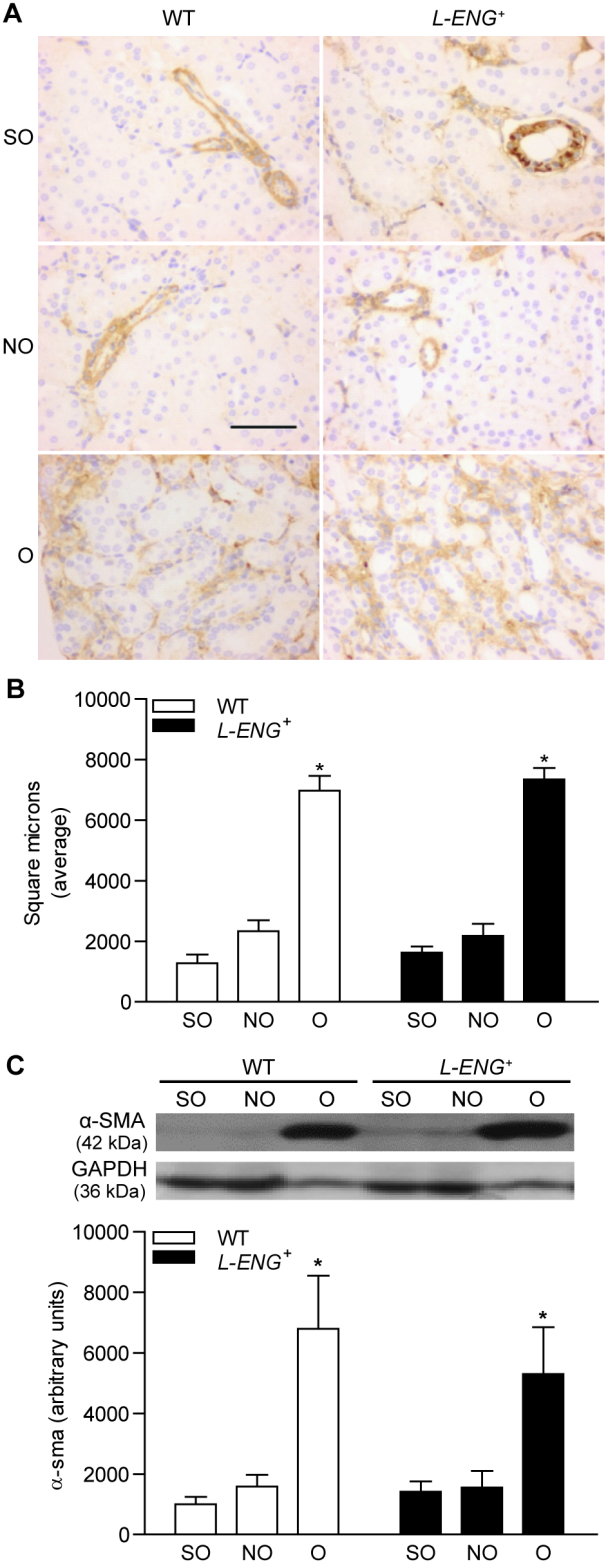
Effect of L-Endoglin overexpression on myofibroblast abundance following ureteral obstruction. Representative immunohistochemistry images for α-SMA (a) in sham operated (SO), non-obstructed (NO) and obstructed (O) kidneys from WT and *L-ENG* ^+^ mice. Bar = 100 µm. (b) Quantification of α-SMA-positive stained area in sham operated (SO), non-obstructed (NO) and obstructed (O) kidneys from WT and *L-ENG* ^+^ mice, expressed as square microns. (c) Western blot analysis of α-SMA protein amount in SO, NO and O kidneys from WT and L-ENG^+^ mice. A representative western blot among 5–7 performed in each group is shown on top. Densitometry analysis is represented as the mean ± SEM of the 5–7 western performed per group. *P<0.01 vs. SO kidneys. #P<0.05 vs. O kidneys from WT mice.

Myofibroblasts are a major source of extracellular matrix proteins and its presence is a hallmark of renal fibrosis [27]. To assess whether the different degree in renal fibrosis between O kidneys of *L-ENG* ^+^ and WT mice was due to differences in myofibroblast abundance, this parameter was evaluated by measuring the content of α-SMA, a specific protein for myofibroblasts [32]. Immunostaining analysis revealed that, whereas α-SMA expression is limited to vessel walls in NO and sham kidneys, a strong expression in the renal interstitium was observed in O kidneys, without apparent differences between O kidneys from *L-ENG* ^+^ and wild type mice (Figure 5a). The quantification of α-SMA-positive stained areas showed a similar increase in both WT and *L-ENG* ^+^ O kidneys. Assuming that the stained area is proportional to the number of myofibroblasts, we can suggest that the increase in myofibroblast number was similar in both groups of animals (Figure 5b). In addition, western blot analysis revealed that O kidneys showed a higher α-SMA expression than NO kidneys, whereas no significant differences in α-SMA content between O kidneys from *L-ENG* ^+^ and WT animals were observed (Figure 5c).

### Effects of L-ENG overexpression in the TGF-β/Smad signalling pathway

**Figure 6:**
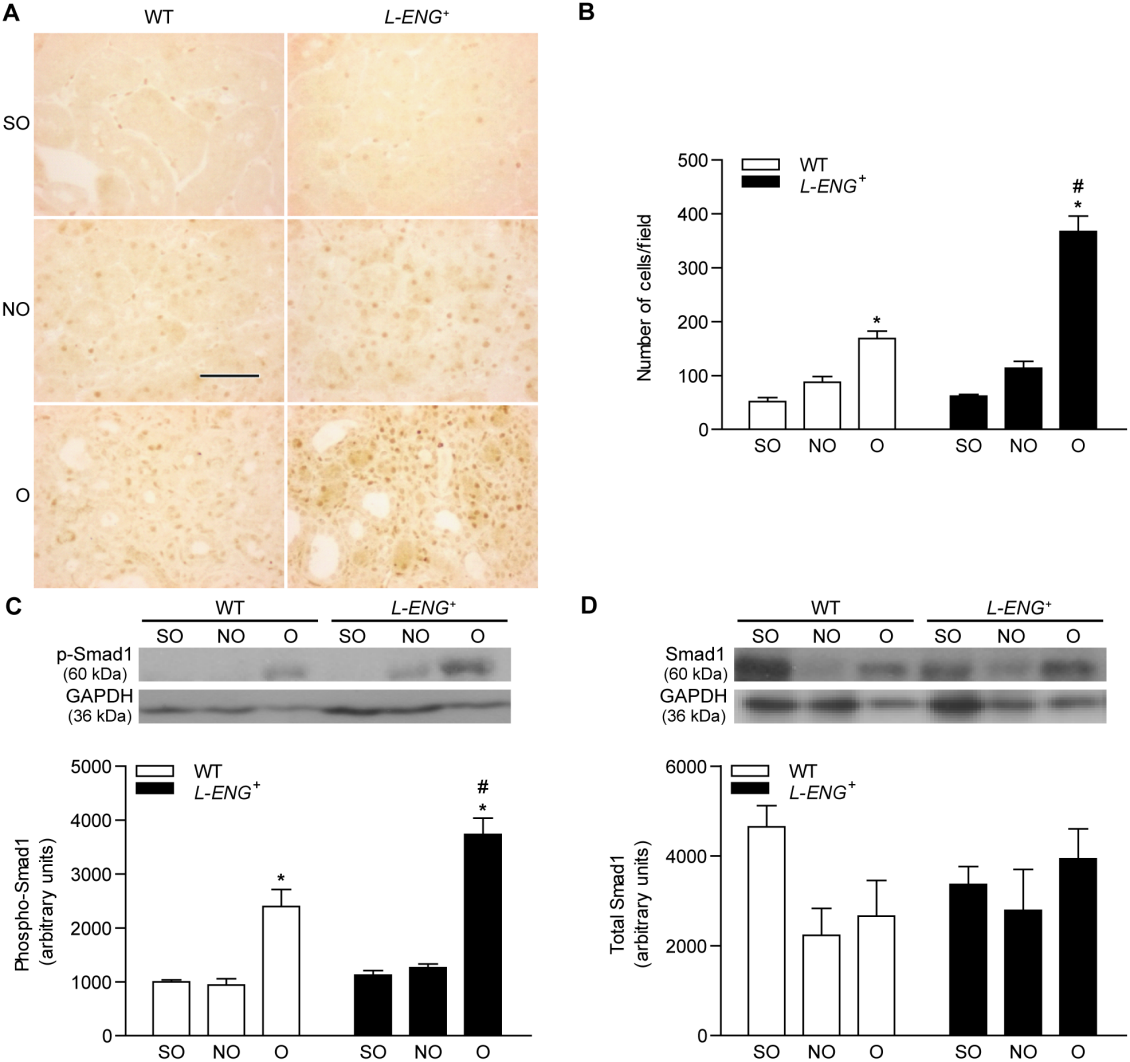
Effect of L-Endoglin overexpression on Smad1 and phospho-Smad1 expression following ureteral obstruction. (a) Representative immunohistochemistry images for phospho-Smad1 in sham operated (SO), non-obstructed (NO) and obstructed (O) kidneys from WT and *L-ENG* ^+^ mice. Bar = 100 µm. (b) Histogram representing the number of phospho-Smad1-positive nuclei per field in SO, NO and O kidneys from WT and *L-ENG* ^+^ mice. Data is represented as mean ± SEM. Western blot analysis of phospho-Smad1 (c) and total Smad1 (d) protein amount in SO, NO and O kidneys from WT and *L-ENG* ^+^ mice. A representative western blot among 5–7 performed in each group is shown on top. Densitometry analysis is represented as the mean ± SEM of the 5–7 western performed per group. *P<0.01 vs. SO kidneys. #P<0.05 vs. O kidneys from WT mice.

**Figure 7:**
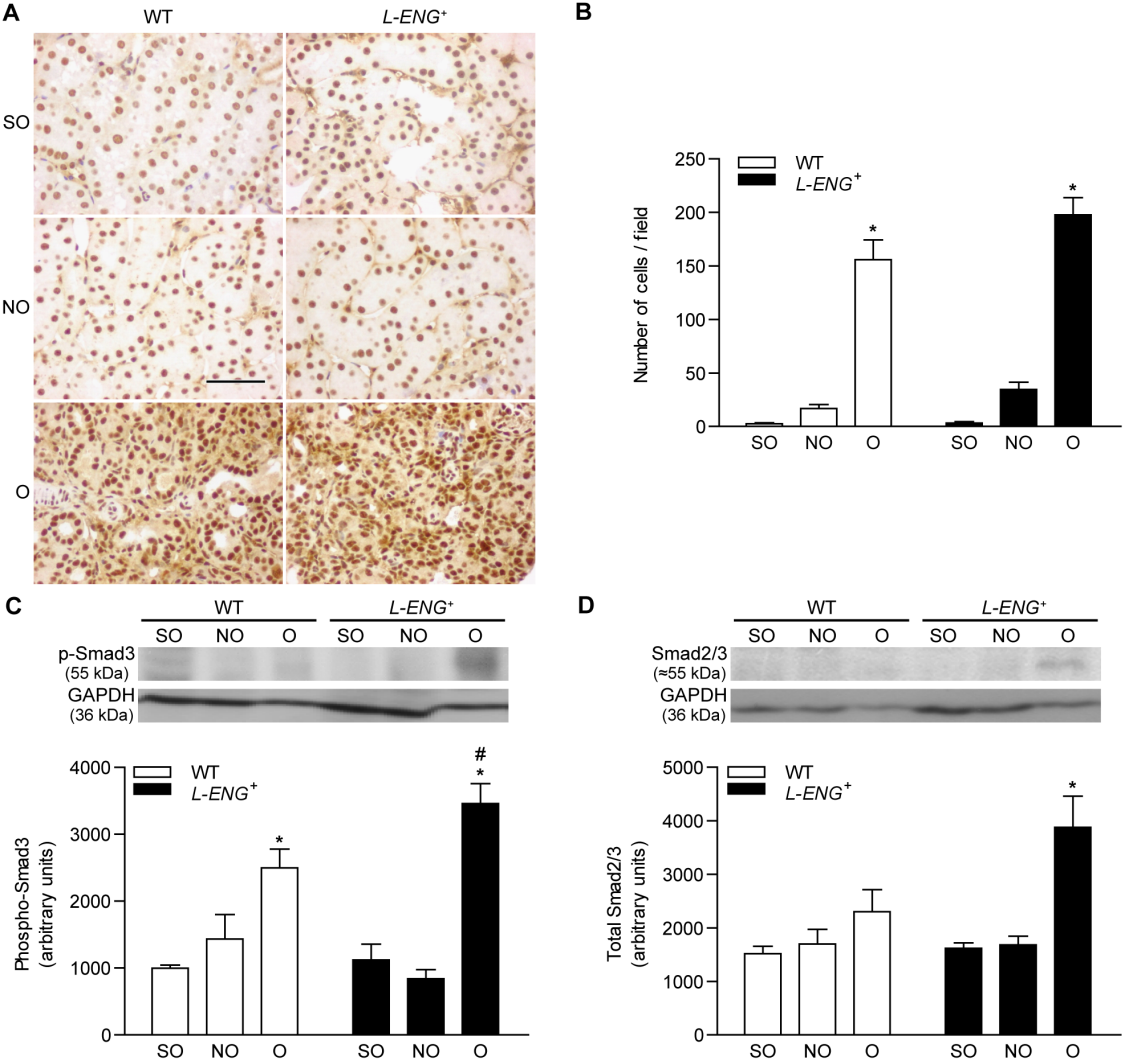
Effect of L-Endoglin overexpression on Smad2/3 and phospho-Smad3 expression following ureteral obstruction. (a) Representative immunohistochemistry images for phospho-Smad3 in sham operated (SO), non-obstructed (NO) and obstructed (O) kidneys from WT and *L-ENG* ^+^ mice. Bar = 100 µm. (b) Histogram representing number of nuclei positively stained for phospho-Smad3 per field in SO, NO and O kidneys from WT and *L-ENG* ^+^ mice. Data is represented as mean ± SEM. Western blot analysis of phospho-Smad3 (c) and total Smad2/3 (d) protein amount in SO, NO and O kidneys from WT and *L-ENG* ^+^ mice. A representative western blot among 5–7 performed in each group is shown on top. Densitometry analysis is of the 5–7 western performed per group. *P<0.01 vs. SO kidneys. #P<0.05 vs. O kidneys from WT mice.

It is well know that TGF-β promotes fibrosis activating specific downstream Smad proteins. To elucidate how *L-ENG* overexpression contributes to increased renal fibrosis, we assessed Smad1 and Smad3 phosphorylation by measuring the abundance of p-Smad1 and p-Smad3 by western blot and immunohistochemistry. Immunohistochemistry for p-Smad1 showed a clear nuclear localization of this protein in both interstitial and tubular nuclei, expression that is mostly detected in O kidneys (Figure 6a). Accordingly, the number of p-Smad1-stained nuclei was higher in *L-ENG* ^+^ than in WT mice (Figure 6b). Sham animals exhibited a weak p-Smad1 signal, circumscribed to nuclei of interstitial cells. Moreover, immunohistochemistry for p-Smad2/3 also revealed a remarkably higher p-Smad2/3 expression in O kidneys as compared with NO and sham kidneys (Figure 7a).

Western blot analysis showed that levels of p-Smad1 and p-Smad3 were higher in O than in NO or sham kidneys, being these levels significantly higher in O kidneys from *L-ENG* ^+^ than in those from WT animals (Figures 6c and 7c). Total Smad2/3 and total Smad1 expression was also assessed by western blot. While there was no difference in total Smad1 content between all the groups (Figure 6d), Smad2/3 levels were higher in O kidneys from *L-ENG* ^+^ than in O kidneys from WT mice (Figure 7d).

### L-Eng overexpression increases ECM production in renal fibroblasts *in vitro*

**Figure 8:**
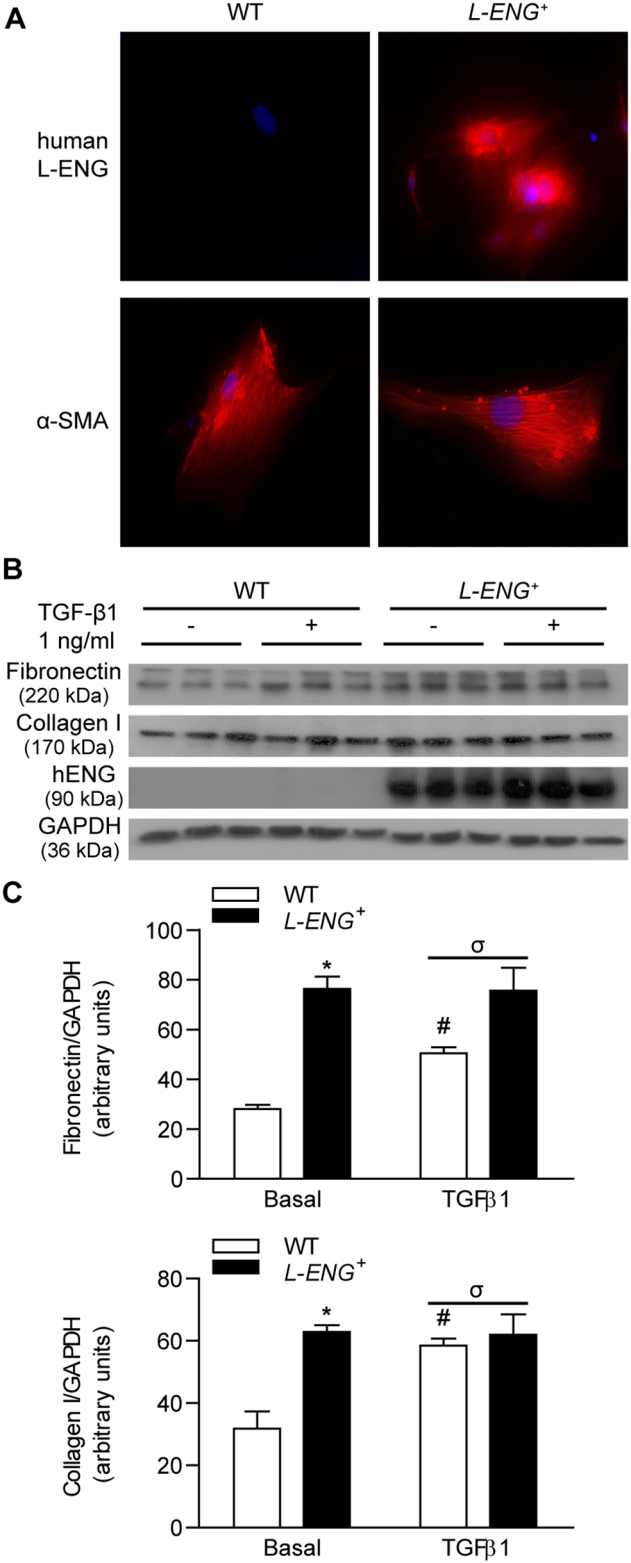
Effect of L-Endoglin overexpression on ECM synthesis in renal fibroblasts. (a) Immunofluorescence of human L-Endoglin and α-SMA in WT and *L-ENG* ^+^ renal fibroblasts. Magnification, 1,000X. Note the presence of human L-endoglin only in L-ENG^+^ renal fibroblasts. (b) Effect of L-Endoglin on ECM protein levels in renal fibroblasts. Representative western blot analysis of fibronectin, collagen I, human L-Endoglin and GAPDH proteins in WT and *L-ENG* ^+^ renal fibroblasts under basal conditions and after TGF-β1 (1 ng/mL) treatment for 24 hours. Densitometric analysis is represented as the mean ± SEM. *P<0.01 vs. WT fibroblasts in basal conditions. ^#^P<0.05 for TGF-β1 treatments vs. basal conditions. ^σ^P<0.05 for TGF-β1 treatment in *L-ENG* ^+^ vs. TGF-β1 treatment in WT fibroblasts.

With the aim to evaluate whether L-Endoglin overexpression influences the activity of fibroblasts, renal fibroblasts from *L-ENG* ^+^ and wild type mice were isolated and cultured. Both *L-ENG* ^+^ and WT-derived fibroblasts were α-SMA positive (Figure 8a). L-ENG was detected in *L-ENG* ^+^−, but not in WT-derived fibroblasts (Figure 8a, b). The fibrogenic potential of *L-ENG* ^+^ fibroblasts was assessed by measuring collagen I and fibronectin production. Western blot analysis revealed that collagen I and fibronectin production was significantly higher in *L-ENG* ^+^ fibroblasts than in wild type fibroblasts (Figure 8b, c). Treatment with 1 ng/mL TGF-β1 induced an increase of collagen I and fibronectin expression in WT fibroblasts. However, this increase was not observed in *L-ENG* ^+^ fibroblasts after treatment with TGF-β1 (Figure 8b, c).

### Effects of L-ENG overexpression on the TGF-β/Smad signalling pathway in renal fibroblasts

**Figure 9:**
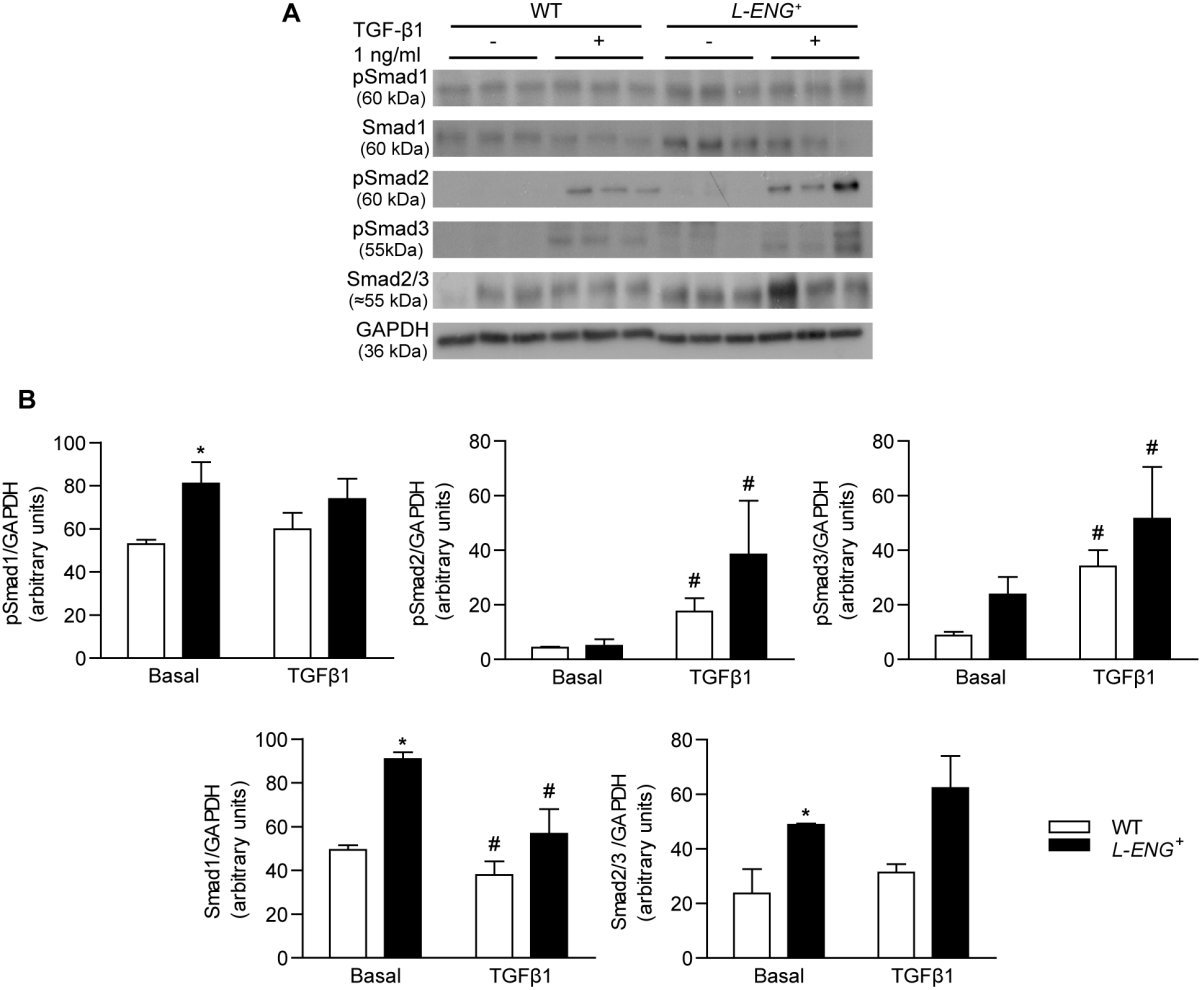
Effect of L-Endoglin overexpression on TGF-β1/Smad signalling in renal fibroblasts. (a) Representative western blot analysis of phospho-Smad1, phospho-Smad2, phospho-Smad3, Smad2/3, Smad1 and GAPDH protein expression in WT and *L-ENG* ^+^ renal fibroblasts under basal conditions and after TGF-β1 (1 ng/mL) treatment for 30 minutes. (b) Densitometric analysis is represented as the mean ± SEM. *P<0.01 vs. WT fibroblasts in basal conditions. ^#^P<0.05 for TGF-β1 treatment vs. basal conditions.

Western blot analysis showed that basal Smad1 and Smad3 phosphorylation were higher in *L-ENG* ^+^ than in WT fibroblasts. Treatment with 1 ng/mL TGF-β1 for 30 min increased Smad2 and Smad3 phosphorylation, but not Smad1 phosphorylation in WT and *L-ENG* ^+^ fibroblasts. Moreover, TGF-β1-induced Smad2 and Smad3 phosphorylation was higher in *L-ENG* ^+^ than in WT fibroblasts (Figure 9a, b).

## Discussion

Our results suggest that L-Endoglin overexpression is associated to increased renal fibrosis after UUO mainly due to the enhanced ability of renal myofibroblasts to synthesize ECM components.

In previous studies, we have observed that endoglin expression was increased in different experimental models of renal fibrosis [11], [33]. Furthermore, endoglin haploinsufficiency does not seem to affect the fibrosis induced in the UUO model [12]. As the expression of the different membrane endoglin isoforms was not assessed in those experiments, and it has been reported that L- and S-Endoglin show distinct modulatory effects on TGF-β signaling [8], [29], the contribution of endoglin and its isoforms to renal fibrosis remains unclear. In this study, our aim has been to evaluate if the overexpression of L-Endoglin, the predominant endoglin isoform, modulates renal fibrosis after UUO. Our results show that both total renal fibrosis, assessed by Masson’s trichrome and Sirius red staining, and expression of collagen I and fibronectin, two major components of ECM, were higher in obstructed kidneys from *L-ENG^+^* than those from WT mice. qPCR analysis showed an increase in both collagen I and fibronectin mRNA expressions after UUO, and this increase was slightly higher in *L-ENG^+^* than in WT mice but the difference was not statistically significant. This can be explained because 15 days after UUO, most of ECM is already synthesized and deposited in the kidney and actual ECM components synthesis is very low.

Myofibroblasts abundance is a major feature of renal fibrosis [1]. Several authors have proposed α-SMA as a reliable myofibroblast marker [34]. Our results show that the amount of α-SMA in the obstructed kidneys, assessed by either WB or immunohistochemistry, was similar in *L-ENG* ^+^ and wild type mice, thus suggesting that the abundance of myofibroblasts was similar in the obstructed kidneys of both strains.

Our results are in agreement with several authors that have shown a profibrotic role of endoglin in different fibrotic contexts such as kidney after irradiation [13] and heart after thoracic aorta constriction [17]. Although a role for endoglin in ECM down regulation had been previously suggested, this conclusion was obtained mainly from *in vitro* studies [9], [10], [29], [35], [36], [37].

TGF-β is able to signal through ALK1 or ALK5, leading to phosphorylation of Smad1/5 or Smad2/3, respectively [4], [29], [36], [38]. It has been observed that UUO induces an increase in Smad2 and Smad3 phosphorylation [39], and the contribution of TGF-β/Smad2/3 pathway in renal fibrosis have been extensively studied [5], [40], [41], [42], [43]. Our results of WB and immunohistochemistry show that Smad3 activation increases in obstructed kidneys from both WT and *L-ENG* ^+^ mice, but this increase is higher in *L-ENG* ^+^ than in WT mice. This result suggests that the increased fibrosis observed in obstructed kidneys from *L-ENG* ^+^ mice may be related with a higher Smad3 phosphorylation. In addition, immunohistochemistry studies revealed that this increase was located in cells placed in the tubular interstitium but not in tubular cells, suggesting that higher Smad2/3 phosphorylation does not seem to be related with changes in epithelial cell function but with changes in interstitial cells, presumably fibroblasts.

The contribution of Smad1/5 pathway in fibrosis is controversial. While several authors have shown that this pathway is antifibrotic when is induced by BMP-7 [44], [45], others have shown a pro-fibrotic role, especially in diabetic nephropathy [46], [47], [48] and also in dermal sclerosis [49]. In this study we have observed that overexpression of L-Endoglin not only potentiates phosphorylation of Smad2/3, but also of Smad1, as phospho-Smad1 increase observed in obstructed kidneys was higher in *L-ENG* ^+^ than in WT mice. Our study shows that increased phospho-Smad1 immunostaining in *L-ENG* ^+^ mice is located in the tubular interstitium, suggesting that L-Endoglin overexpression is associated with Smad1 activation in renal myofibroblasts, and that the activation of this pathway may enhance ECM protein synthesis by these cells, as postulated by some authors [14], [49].

Cultured renal myofibroblasts overexpressing L-Endoglin synthesize more collagen I and fibronectin under basal conditions, probably due to a higher Smad1, Smad2 and Smad3 phosphorylation in these conditions. However, treatment with TGF-β1 (1 ng/mL) induced an increase of collagen type I and fibronectin in wild type, but not in *L-ENG* ^+^ renal fibroblasts. This lack of TGF-β1-induced fibrotic response may be due to the higher Smad2 phosphorylation observed in *L-ENG* ^+^ fibroblasts. Antifibrotic role of Smad2 activation has been described in Smad2 KO mouse embryo fibroblasts [50].

In conclusion, we have shown that overexpression of L-Endoglin increases renal fibrosis following UUO, suggesting its active participation in this pathological process. This effect may be explained by a higher L-Endoglin-dependent Smad1 and Smad3 phosphorylation. The increase in renal fibrosis observed in *L-ENG* ^+^ mice is not due to a higher number of myofibroblasts, but a higher ability of myofibroblasts in the kidney of these animals to synthesize ECM proteins.

## Supporting Information

Figure S1 **Human L-Endoglin expression in different tissues.** Expression of human L-Endoglin (hENG) in heart, spleen, lung, liver and kidney tissues from *L-ENG^+^* mice. No expression of the protein is detected in WT mice. Human lung (A) and human cervical paraganglioma (B) biopsies were used as positive control of L-Endoglin expression. GAPDH was used as load control.
(TIF)

Figure S2 **Effect of unilateral ureteral obstruction in renal cortex and medulla thickness.** Low amplification images of non-obstructed (NO) and obstructed kidneys (O) stained with (a) hematoxilin-eosin and (b) Masson’s trichrome. Blue line: thickness of the cortex. Black bar: 1 mm. Note the marked decrease in cortex and medulla thickness in O kidneys.
(TIF)

Figure S3 **Effect of L-Endoglin overexpression on collagen Iα and fibronectin mRNA expression after unilateral ureteral obstruction.** mRNA for collagen Iα (a) and fibronectin (b) in sham operated (SO), non-obstructed (NO) and obstructed (O) kidneys from WT and *L-ENG* ^+^ mice were analyzed by RT-PCR. PRS13 was used as housekeeping gene. Number of mice in each group: SO (n = 3); NO (n = 4); O (n = 4). *P<0.05 vs. their respective SO kidneys.
(TIF)
