# Supplementary figures and images for: L-Endoglin Overexpression Increases Renal Fibrosis after Unilateral Ureteral Obstruction
Source: PLoS One. 2014 Oct 14;9(10):e110365. doi: 10.1371/journal.pone.0110365 (PMC4196986; doi:10.1371/journal.pone.0110365)

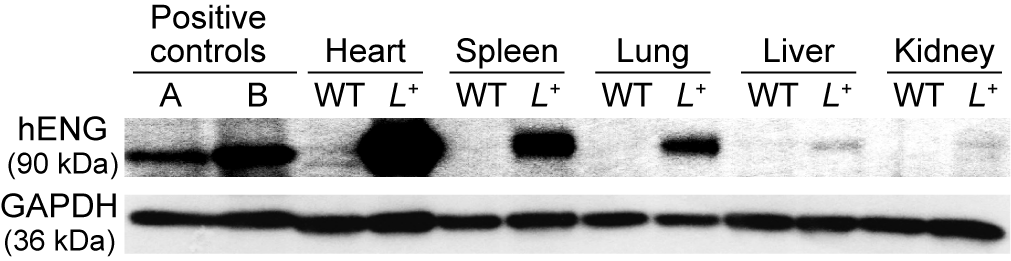

Supplement: Figure S1 — Human L-Endoglin expression in different tissues. Expression of human L-Endoglin (hENG) in heart, spleen, lung, liver and kidney tissues from L-ENG+ mice. No expression of the protein is detected in WT mice. Human lung (A) and human cervical paraganglioma (B) biopsies were used as positive control of L-Endoglin expression. GAPDH was used as load control. (TIF) [file pone.0110365.s001.tif]

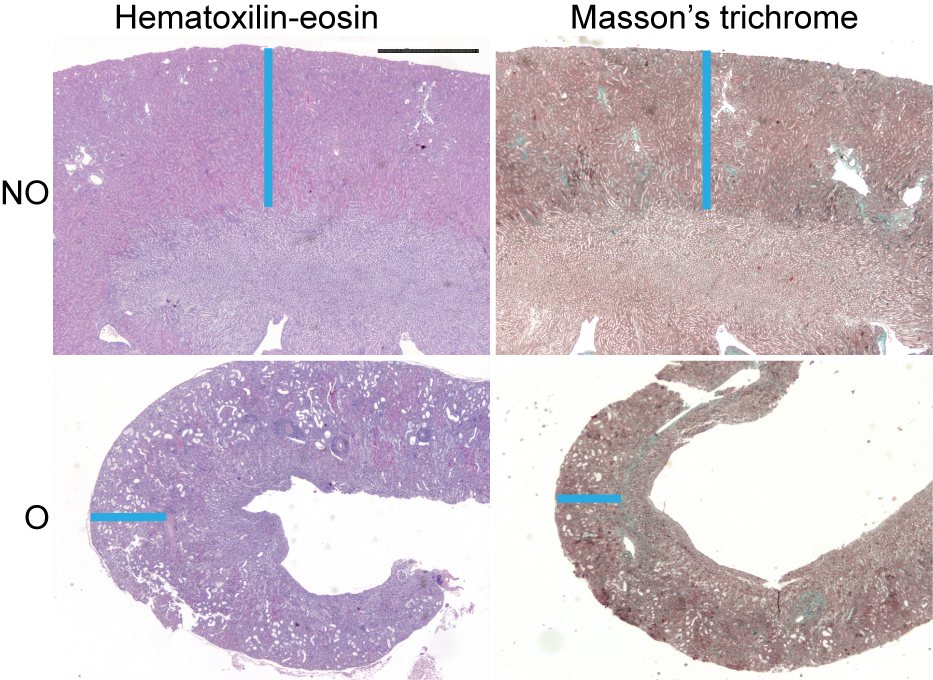

Supplement: Figure S2 — Effect of unilateral ureteral obstruction in renal cortex and medulla thickness. Low amplification images of non-obstructed (NO) and obstructed kidneys (O) stained with (a) hematoxilin-eosin and (b) Masson’s trichrome. Blue line: thickness of the cortex. Black bar: 1 mm. Note the marked decrease in cortex and medulla thickness in O kidneys. (TIF) [file pone.0110365.s002.tif]

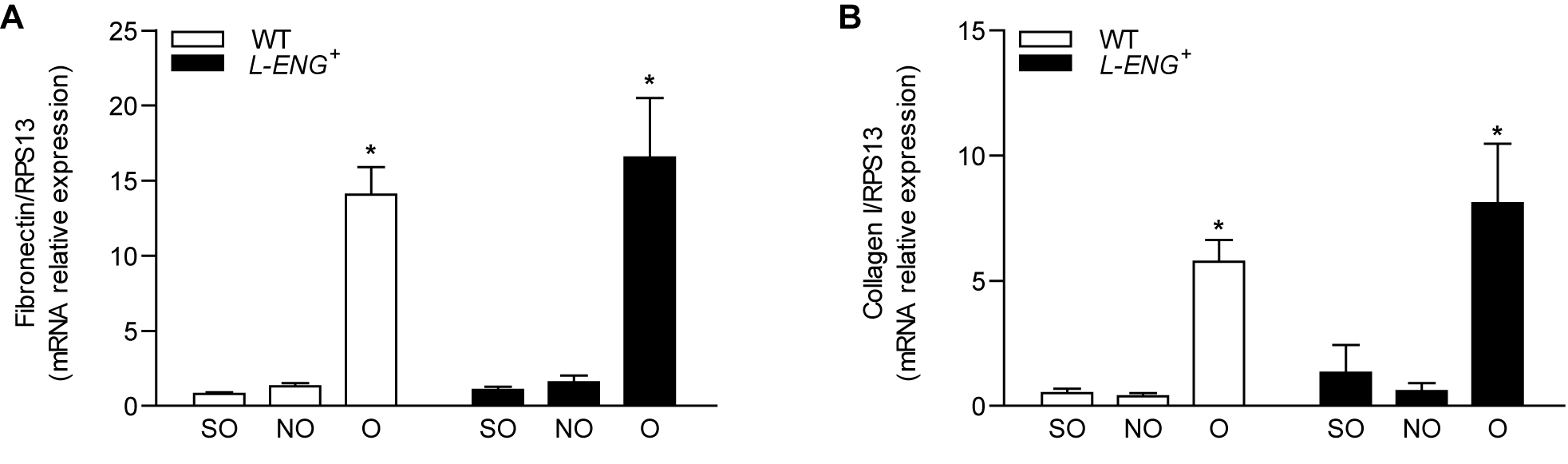

Supplement: Figure S3 — Effect of L-Endoglin overexpression on collagen Iα and fibronectin mRNA expression after unilateral ureteral obstruction. mRNA for collagen Iα (a) and fibronectin (b) in sham operated (SO), non-obstructed (NO) and obstructed (O) kidneys from WT and L-ENG + mice were analyzed by RT-PCR. PRS13 was used as housekeeping gene. Number of mice in each group: SO (n = 3); NO (n = 4); O (n = 4). *P<0.05 vs. their respective SO kidneys. (TIF) [file pone.0110365.s003.tif]
